# Management of Exciton Distribution for High-Performance Organic Light-Emitting Diodes Based on Interfacial Exciplex Architecture

**DOI:** 10.3390/molecules28207028

**Published:** 2023-10-11

**Authors:** Ren Sheng, Cong Chen, Erdong Zhang, Wencheng Zhao, Jihua Tang, Duxu Yan, Zhengze Li, Ping Chen

**Affiliations:** 1 Institute of Physics and Electronic Information, Yantai University, Yantai 264005, China; rensheng@ytu.edu.cn (R.S.); congchen@s.ytu.edu.cn (C.C.); erdongzhang@s.ytu.edu.cn (E.Z.); 18265892850@163.com (W.Z.); fanhuokua111@126.com (J.T.); yandx316@s.ytu.edu.com (D.Y.); 2 State Key Laboratory on Integrated Optoelectronics, College of Electronic Science & Engineering, Jilin University, Changchun 130012, China; 13091682837@163.com

**Keywords:** organic light-emitting diodes, exciplex, phosphorescence, high efficiency

## Abstract

Interfacial exciplex has recently been adopted as an effective host to achieve phosphorescent organic light-emitting diodes (OLEDs) with high efficiencies and low driving voltages. However, a systematic understanding of exciton recombination behavior in either host of interfacial exciplex is still deficient. Herein, the strategic design rule of interfacial exciplex host is proposed to overcome the negative effects of direct trapping recombination by systematically investigating exciton recombination behavior in interfacial exciplex hosts. As a result, blue and orange phosphorescent devices acquire peak external quantum efficiencies of 23.5% and 29.2% with low turn-on voltages. These results provide a simple method to realize highly efficient OLEDs aiming for general lighting and display applications.

## 1. Introduction

Organic light-emitting diodes (OLEDs) hold great promise for applications in full-color display and solid-state lighting because of their striking advantages, such as high efficiency, fast response, and flexibility [1,2,3,4]. In order to pursue high efficiency, phosphor-doped OLEDs are a highly effective approach because of 100% internal quantum efficiency in principle via harnessing both singlet and triplet excitons [5,6,7]. Generally, phosphorescent dyes are used as the guest and doped into the host matrix to realize a high performance of energy transfer system [8,9]. Consequently, the performance of devices is mainly determined by the properties of the host materials, which may act as the main charge transport channels and recombination centers, resulting in increased limitations in fabrication [10,11,12,13].

Recently, the exciplex system co-doped between the donor and acceptor is considered a promising technology to overcome the above barriers [14,15,16,17]. Owing to the intrinsically small energy gap (ΔE_ST_) between the triplet level (T_1_) and singlet level (S_1_), triplet excitons could be upconverted into singlet by efficient reverse intersystem crossing (RISC) process [18,19,20]. This efficient upconversion provides markedly enhanced long-range dipole–dipole coupling (Förster energy transfer) from the exciplex host to dopants, leading to high-efficiency OLEDs under low doping levels [21,22]. However, the co-evaporation of mixed host materials with dopant significantly aggravates the complexity of the manufacturing process, obstructing the large-scale commercialization application.

To settle this problem, interfacial exciplex is exploited as a host for achieving high-performance OLEDs with an uncomplicated fabrication process [23,24]. By employing bilayer exciplex architecture, charge-transfer (CT) excitons could be formed between the donor and acceptor at the heterointerface conspicuously with the suppressed formation of the donor excited states, leading to an efficient energy transfer from host to guest [24]. Recently, Wu et al. demonstrated orange OLED with a high external quantum efficiency (EQE) of 14.3% and suppressed roll-off by combining an ultrathin phosphor layer and interfacial exciplex host [25]. Wang et al. reported efficient interfacial-exciplex-hosted red phosphorescent OLED with maximum power efficiency (PE) of 40.4 lm W^−1^ by solution-processing method [26]. However, the systematic investigation of the EL mechanism in either host of interfacial exciplex has not yet been demonstrated, and OLED based on an interfacial exciplex system with outstanding efficiency is scarcely reported so far.

In this work, the influence of exciton recombination behavior in interfacial exciplex hosts on the performance of the device is investigated, indicating suppressed charge trapping and efficient Förster energy transfer from interface exciplex to the guests are the key factors for improving the performance of interfacial exciplex hosted devices. By doping phosphorescent dyes into the acceptor host of interface exciplex structure constructed by the donor of 2,6-bis(3-(carbazol-9-yl)phenyl)pyridine (26DCzPPy) and the acceptor of 4,6-bis[3,5-(dipyrid-3-yl)-phenyl]-2-phenylpyrimidine (B4PyPPM), highly efficient blue and orange phosphorescent OLEDs with maximum EQEs of 23.5% and 29.2% are provided, which is one of the best values in OLEDs based on simplified architectures.

## 2. Results

**Figure 1:**
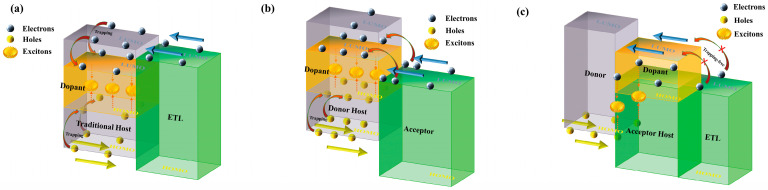
The operational mechanism of devices based on (**a**) traditional host, (**b**) donor host, and (**c**) acceptor host.

As for common phosphorescent OLEDs, hole-dominated or bipolar organic materials are frequently used as hosts for dopants according to the design principle of charge balance [27,28,29]. As depicted in Figure 1a, the highest occupied molecular orbital (HOMO) levels of the hosts are substantially below the HOMO of the dopants due to the smaller band gap of dopants; thus, the dopants are expected to trap holes. On the other hand, electrons could transport to the lowest unoccupied molecular orbital (LUMO) of hosts, easily arising from a minor energy gap between the electron-transport layers and hosts. These electrons are further trapped by dopants, leading to dominated trap-assisted charge recombination on dopant molecules. While referring to donor-hosted devices based on interfacial exciplex, as shown in Figure 1b, owing to large HOMO-LUMO offset between donor and acceptor, electrons accumulated at the heterointerface prefer to overcome barriers and inject into the dopants rather than donor molecules, causing increased operation voltage. Specifically, the charge trapping effect would hinder the transport of holes inside the emission layers; therefore, holes could barely reach the heterointerface, resulting in the significantly suppressed formation of CT excitons. In such cases, efficient energy transfer from exciplex host to guest is dramatically restrained, which is the main reason for poor EL performance of the devices with interfacial exciplex host. In contrast, by using acceptors as hosts, the trap-assisted recombination on dopants could be suppressed, as shown in Figure 1c. Thanks to the LUMO energy barrier between acceptor molecules and dopants, electrons would pass through the acceptor layer along the LUMO of the acceptor predominantly without a trapping effect. Note that CT excitons could be effectively formed by interfacial molecular interactions between donor and acceptor. Accordingly, it is possible that the energy of the CT excitons would be transferred to the dopants efficiently, leading to superior device performance.

**Figure 2:**
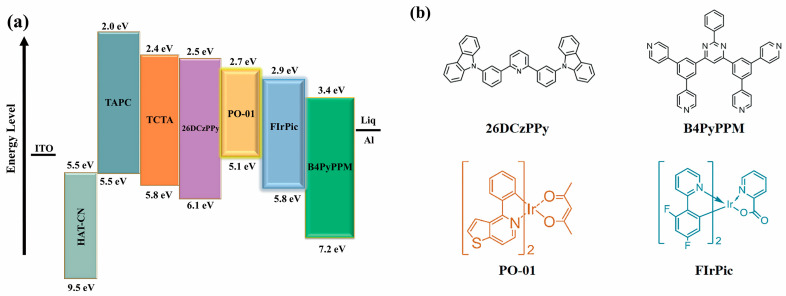
(**a**) The detailed energy level diagram of the used materials. (**b**) The molecular structures of the materials used in this work.

**Figure 3:**
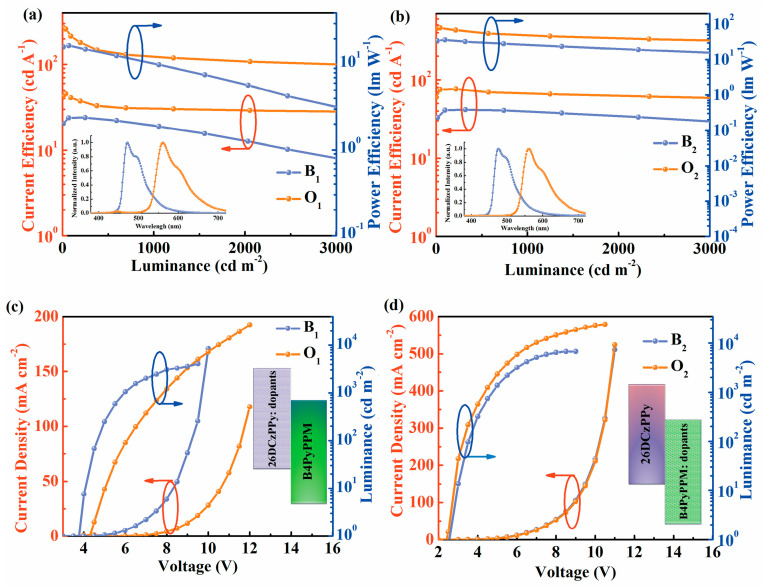
The current efficiency-luminance-power efficiency characteristics of devices (**a**) B_1_–O_1_ and (**b**) B_2_–O_2_. Inserts: normalized EL spectra of the devices at 5 V. The current density-voltage-luminance characteristics of devices (**c**) B_1_–O_1_ and (**d**) B_2_–O_2_.

**Figure 4:**
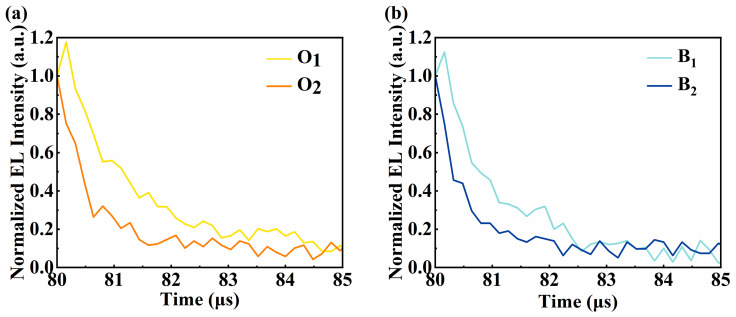
The transient EL decay of (**a**) devices O_1_ and O_2_ and (**b**) devices B_1_ and B_2_.

To demonstrate the influence of different operating mechanisms on device performance, two groups of devices are fabricated based on the interfacial exciplex formed by 26DcZPPy and B4PyPPM with the structure of ITO/HAT-CN (10 nm)/TAPC (35 nm)/TCTA (5 nm)/26DcZPPy (5 nm)/emission layer (20 nm)/B4PyPPM (35 nm)/Liq (0.8 nm)/Al. Here, the emission layers are 26DCzPPy: 10%FIrPic; 26DCzPPy: 3%PO-01; B4PyPPM: 10%FIrPic; and B4PyPPM: 3%PO-01, corresponding to device B_1_, O_1_, B_2_ and O_2_, respectively. Figure 2a,b exhibit the detailed energy level diagram and the molecule structures of required materials, respectively. The formation of exciplex could be demonstrated by the red-shift PL spectrum of mixed film compared with those of constituting molecules and TADF characteristics in transient PL decayed curves in Figure S1 [30]. The exciplex of 26DCzPPy: B4PyPPM possesses a minor ΔE_ST_ of 35 meV and an efficient RISC process, which are beneficial for preparing efficient OLEDs [31]. Figure 3a,b show the current efficiency (CE) and PE curves of four devices. Device B_1_ and device O_1_ only give maximum efficiencies of 16.7 lm W^−1^ (24.3 cd A^−1^) and 26.6 lm W^−1^ (45.3 cd A^−1^), respectively, whereas it is found that ultra-high efficiencies are realized as the dopants are doped in B4PyPPM, displaying high PEs of 36.7 lm W^−1^ for device B_2_ and 78.9 lm W^−1^ for device O_2_, which is 2.2 folds higher than that of device B_1_ and 2.9 folds higher than that of device O_1_, respectively. Additionally, devices B_2_ and O_2_ show low turn-on voltages of 2.6 V and 2.4 V despite large charge injection barriers between 26DcZPPy and B4PyPPM, which are significantly reduced compared to 3.8 V for device B_1_ and 4.2 V for O_1_. It can be considered that the properties of host materials greatly affect the EL performance of devices. Despite the bipolar transport property of 26DcZPPy, B4PyPPM is adequately demonstrated to be the host for emitting dyes in our interface exciplex system. It is generally agreed that the charge transport pathway is a typical behavior to estimate the emission mechanism of the devices [32]. As can be seen in Figure 3c,d, current densities show a strong dependence on dopants in 26DCzPPy hosted devices, which have little effect on current densities in B4PyPPM hosted devices. These phenomena possibly indicate that trap-assisted recombination plays an important role in the EL process of the devices with 26DCzPPy host, which is suppressed in the devices with B4PyPPM host. To further understand the emission mechanism, transient EL decay curves are further carried out. As shown in Figure 4, transient overshoots in the turn-off region are observed in devices O_1_ and B_1_ with the 26DcZPPy-hosted structure, which can be attributed to charge carrier trapping in the dopants [33]. However, overshoots are barely observed in devices O_2_ and B_2_. This phenomenon demonstrates that accumulated charge carriers are released and recombined after bias turn-off in devices O_1_ and B_1_, which may also cause longer decay time in devices O_1_ and B_1_ compared to those in devices O_2_ and B_2_.

**Figure 5:**
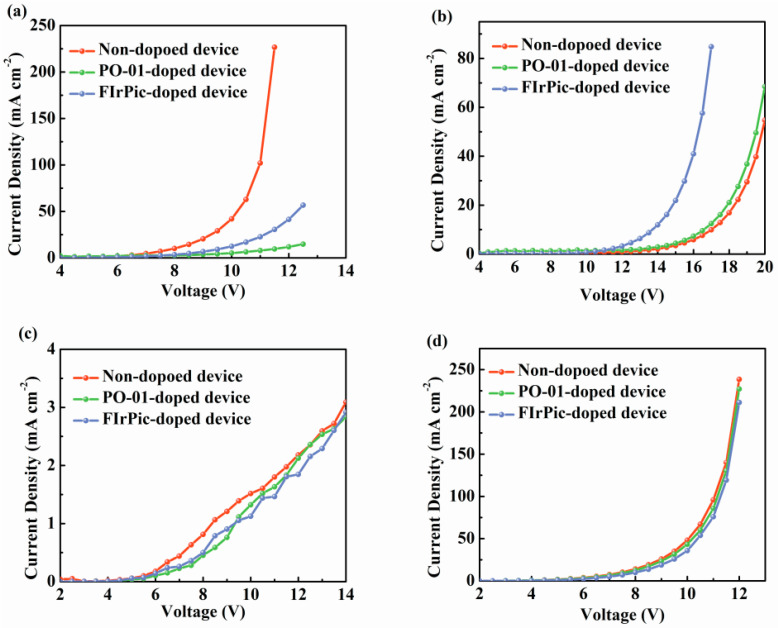
The current density-voltage characteristics of (**a**) hole- and (**b**) electron-only devices based on 26DcZPPy hosted devices. The current density-voltage characteristics of (**c**) hole- and (**d**) electron-only devices based on B4PyPPM hosted devices.

To further demonstrate the influence of dopants on charge transport properties in devices with interfacial exciplex host, single-carrier devices are carried out successively with the structure of ITO/HAT-CN (10 nm)/TAPC (35 nm)/TCTA (5 nm)/26DcZPPy (5 nm)/26DCzPPy: 10%FIrPic or 3%PO-01 (20 nm)/TAPC (35 nm)/HAT-CN (10 nm)/Al and ITO/Liq (0.8 nm)/B4PyPPM (35 nm)/B4PyPPM: 10%FIrPic or 3%PO-01 (20 nm)/B4PyPPM (35 nm)/Liq (0.8 nm)/Al. Figure 5a,b show the single-carrier current density–voltage characteristics of 26DcZPPy hosted devices. It is important to confirm that doped devices exhibit significantly decreased hole current densities compared to that of the non-doped device, suggesting the dopants may act as charge trapping centers for holes due to the shallower HOMO levels of dopants than that of 26DcZPPy. Nevertheless, the electron current densities of doped devices show huge enhancements compared to the doping-free device, which can be explained by the fact that electrons transported from the B4PyPPM layer preferably inject into the LUMO levels of dopants assigned to their deeper LUMO levels compared to that of 26DCzPPy, which could facilitate electron transport into the emission layer. The higher electron current in the FIrPic-doped device than in PO-01 is attributed to the deeper LUMO and dominant electron transport property of FIrPic molecules. Figure 5c,d show the single carrier current density–voltage characteristics of B4PyPPM-hosted devices. Obviously, there is no remarkable change in hole and electron current between doped and doping-free devices, indicating that dopants show barely any impact on both hole and electron transport, which is consistent with the above results.

**Figure 6:**
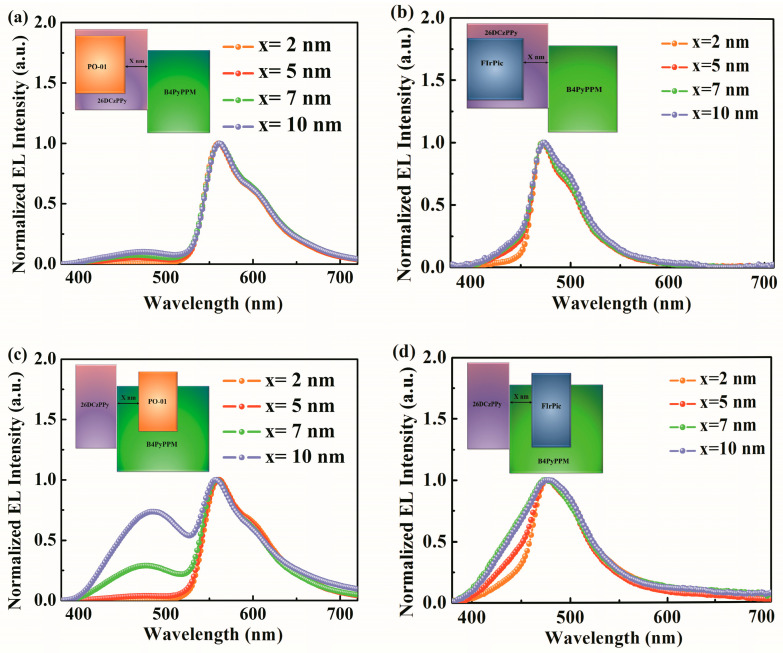
The normalized EL spectra of devices based on the emission layer of (**a**) 26DCzPPy: 3%PO-01, (**b**) 26DCzPPy: 10%FIrPic, (**c**) B4PyPPM: 3%PO-01, and (**d**) B4PyPPM: 10%FIrPic at 8 V.

To better comprehend the two types of emission mechanisms, neat spacer layers with different thicknesses are inserted at the 26DcZPPy/B4PyPPM heterointerface in devices based on 26DcZPPy and B4PyPPM hosts, respectively. The emission layer structures of the devices are shown in the insets of Figure 6. The normalized EL spectra of 26DcZPPy hosted devices are shown in Figure 6a,b. All the spectra exhibit major dopant emission along with faint exciplex emission, suggesting the exciplex formation is suppressed at the interface of 26DcZPPy/B4PyPPM due to the hole-trapping effect in the 26DcZPPy host. As a result, the exciton recombination region should be sited in the doped emission layer, which deviates far from the interface of 26DcZPPy/B4PyPPM, leading to the restrained formation of interface exciplex. By contrast, for B4PyPPM hosted devices, holes injected from the anode can easily across the transport layer and accumulate at the 26DcZPPy/B4PyPPM heterointerface, contributing to the large HOMO offset of 1.1 eV between 26DcZPPy and B4PyPPM as well as inferior hole transport property of B4PyPPM. On the other hand, because of the much deeper LUMO of B4PyPPM than that of dopants, electrons trapped by dopant molecules can be negligible. In this case, electrons injected from the cathode can reach the heterointerface without limitation, increasing the generation probability of exciplex-type excitons. Figure 6c,d exhibits the normalized EL spectra of B4PyPPM-hosted devices. As x is less than 5 nm, the interface exciplex generated by interfacial molecular interactions transfers the energy to the dopants effectively, leading to serious quenching of exciplex emission. With the further increasing thickness of the spacer, the blue emission from exciplex increases quickly, indicating energy transfer from exciplex to dopant become less efficient due to the separation between the doped layer and exciton recombination region. It is notable that strong dopant emission can be obtained in B4PyPPM hosted devices even with 10 nm spacers, as shown in Figure 6c,d, suggesting Förster energy transfer could be identified as the main energy transfer channel since the Dexter energy transfer radius is estimated to be 1 nm.

**Figure 7:**
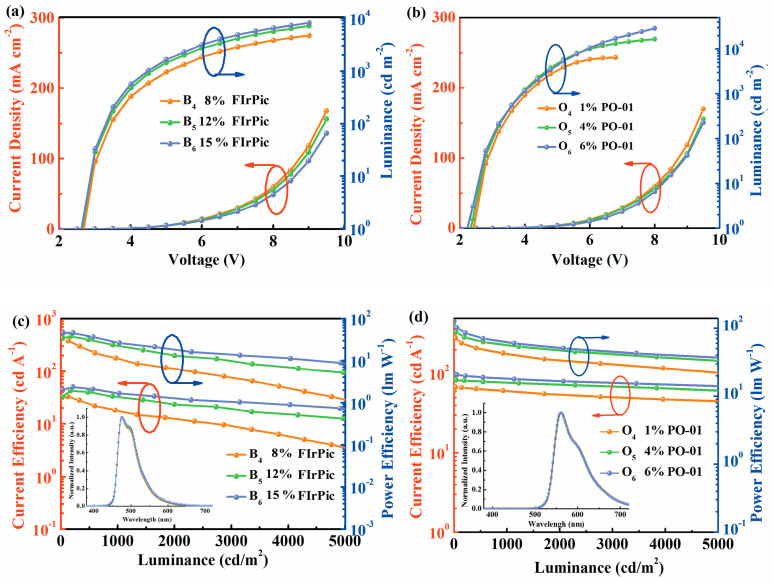
The current density-voltage-luminance characteristics of (**a**) B_4_–B_6_ and (**b**) O_4_–O_6_. The current efficiency-luminance-power efficiency characteristics of (**c**) B_4_–B_6_ and (**d**) O_4_–O_6_ inserts show normalized EL spectra of the devices at 5 V.

To further optimize the performance of the blue and orange devices based on B4PyPPM hosted structure, the devices are fabricated by modulating doping concentration with the structure of ITO/HAT-CN (10 nm)/TAPC (35 nm)/TCTA (5 nm)/26DcZPPy (5 nm)/emission layer (20 nm)/B4PyPPM (35 nm)/Liq (0.8 nm)/Al. Here, the emission layers are B4PyPPM: 8%FIrPic, B4PyPPM: 12%FIrPic, B4PyPPM: 15%FIrPic, B4PyPPM: 1%PO-01, B4PyPPM: 4%PO-01 and B4PyPPM: 6%PO-01, corresponding to device B_4_, B_5_, B_6_, O_4_, O_5_ and O_6_, respectively. As shown in Figure 7a,b, the current density shows a slight variation as the doping concentration rises in both blue and orange devices, demonstrating charge trapping effects can be negligible. It is notable that device B_6_ with 15% FIrPic and O_6_ with 6% PO-01 show low turn-on voltages of 2.6 V and 2.3 V, which are close to the optical energy gaps of their molecules, suggesting the barrier-free exciton recombination in two devices. With the doping level increasing, efficiencies of blue and orange series devices enhance obviously, as shown in Figure 7c,d and Table 1. The maximum PEs of 45.9 lm W^−1^ and 125.9 lm W^−1^ are achieved in devices B_6_ and O_6_, respectively, which are one of the best reported phosphorescent OLEDs as shown in Table 2. Remarkably, even suffering low doping levels in devices B_4_ and O_4_, the energy of exciplex excitons can be harvested by dopants via Förster energy transfer effectively, performing high PEs of 33.6 lm W^−1^ for device B_4_ and 77.9 lm W^−1^ for device O_4_ with undetectable exciplex emission.

Subsequently, for a comparison, blue and orange devices based on 26DcZPPy: B4PyPPM (1:1) co-doped exciplex host are designed, corresponding to device B_3_ and O_3_, respectively. Detailed EL characteristics are shown in Table 1 and Figure S2. The maximum PEs of the two devices are 38.9 lm W^−1^ for device B_3_ and 87.7 lm W^−1^ for device O_3_, which are lower than those of devices B_6_ and O_6_ but significantly better than devices B_1_ and O_1_. It is reasonable that energy transfer between exciplex and dopants should be effective in these devices, whereas the negative effects assigned to non-ignorable charge trapping are still inevitable during the transport process in the emission layer, which results in degradation of device performance.

## 3. Experimental Section

All the devices were fabricated on pre-patterned indium tin oxide (ITO) coated glass, and the sheet resistance of the transparent anode was 20 Ω/square. The ITO substrate was scrubbed sequentially with acetone, ethanol, and deionized water and cleaned in a UV ozone chamber for 5 min before being loaded into a high vacuum thermal system. An amount of 10 nm of 8-hydroxyquinolinolato-lithium (HAT-CN) was used as a hole injection layer. An amount of 1,1-Bis[(di-4-tolylamino)phenyl]cyclohexane (TAPC) and Tris(4-carbazoyl-9-ylphenyl)amine (TCTA) with high hole mobility were used as the hole transport layer and electron blocking layer, respectively. Additionally, 26DCzPPy and B4PyPPM were used as donors and acceptors of the interfacial exciplex, respectively. Bis(4-phenylthieno[3,2-c]pyridinato-N,C2′) acetylacetonate iridium(III) (PO-01) and Bis[2-(4,6-difluorophenyl)pyridinato-C2,N](picolinato)iridium(III) (FIrPic) were used as orange and blue phosphorescent emission dyes, respectively. An amount of 0.8 nm Liq and 100 nm Al were deposited sequentially as cathodes. All the devices were fabricated in a high vacuum (5 × 10^−4^ Pa) thermal evaporation chamber. Organic layers were grown at the rate of 0.1–0.2 nm/s, while PO-01, FIrPic, and Liq were deposited at the rate of 0.02–0.08 Å/s, and Al cathode was deposited at the rate of 0.5 nm/s. Electroluminescence (EL) information of the devices was simultaneously measured using a PR655 spectro-scan spectrometer. The current-voltage and luminance-voltage characteristics were recorded by combining the spectrometer with a programmable Keithley 2450 voltage-current source.

## 4. Conclusions

In summary, highly efficient phosphorescent OLEDs based on simplified architecture are successfully achieved by employing an interface exciplex system constructed by 26DcZPPy and B4PyPPM. The resulting blue and orange phosphorescent OLEDs show maximum EQEs of 23.5% and 29.2% with low turn-on voltages. By investigating exciton recombination behavior in control devices, superior performance can be attributed to the efficient Förster energy transfer and suppressed charge carriers trapping effect via interface exciplex system. We believe that this design concept can provide a promising method for developing highly efficient OLEDs with simplified configurations.

## Figures and Tables

**Table 1:** EL Performance of orange and blue OLEDs.

	V_on_	CE(cd/A)_max/100/1000_	PE(lm/W)_max/100/1000_	EQE(%)_max/100/1000_	CIE
Device B_3_	2.7	38.7/33.9/30.4	38.9/25.7/16.7	18.0/15.8/14.2	(0.16, 0.36)
Device B_4_	2.7	33.0/32.6/17.8	33.6/32.1/11.2	15.3/15.1/8.3	(0.15, 0.36)
Device B_5_	2.6	42.6/37.7/34.0	38.2/36.0/22.8	19.8/17.5/15.8	(0.15, 0.36)
Device B_6_	2.6	50.6/45.7/37.7	45.9/45.4/28.2	23.5/21.2/17.5	(0.15, 0.36)
Device O_3_	2.7	92.2/90.8/83.9	87.7/77.8/51.6	26.9/26.4/24.5	(0.49, 0.50)
Device O_4_	2.4	67.6/59.8/50.0	77.9/66.3/40.5	20.5/17.6/15.1	(0.49, 0.50)
Device O_5_	2.3	85.2/77.7/69.2	107.1/84.3/56.3	24.9/22.7/20.2	(0.49, 0.50)
Device O_6_	2.3	100.2/86.0/76.1	125.9/96.9/61.6	29.2/25.1/22.2	(0.49, 0.50)

V_on_: Turn-on voltages of the devices. CE_max/100/1000_: the maximum CE/CE at 100 cd m^22122^/CE at 1000 cd m^−2^. PE_max/100/1000_: the maximum PE/PE at 100 cd m^−2^/PE at 1000 cd m^−2^. EQE_max/100/1000_: the maximum EQE/EQE at 100 cd m^−2^/EQE at 1000 cd m^−2^. CIE: CIE coordinates at 1000 cd m^−2^. Oversight.

**Table 2:** Summary of EL performance of phosphorescent OLEDs.

		V_on_	PE_max_	CE_max_	EQE_max_
Orange OLEDs	O_6_	2.3	125.9	100.2	29.2
	Ref [25]	3.6	37.4	40.5	14.3
	Ref [34]	3.0	79.4	80.9	26.1
	Ref [32]	2.5	57.6	50.7	18.5
	Ref [35]	2.5	74.1	59.0	20.4
Blue OLEDs	B_6_	2.6	45.9	50.6	23.5
	Ref [36]	2.9	44.7	42.5	-
	Ref [37]	3.0	30.9	32.6	16.0
	Ref [18]	2.6	47.4	40.7	20.1
	Ref [38]	2.62	27.30	29.24	-
	Ref [39]	2.4	66.0	-	30.3

V_on_: Turn-on voltages of the devices. CE_max_: the maximum CE. PE_max_: the maximum PE. EQE_max_: the maximum EQE.

## Data Availability

Not applicable.

## Supplementary Materials

The following supporting information can be downloaded at: https://www.mdpi.com/article/10.3390/molecules28207028/s1. Figure S1. (a) The normalized PL spectra of 26DCzPPy, B4PyPPM and 26DCzPPy: B4PyPPM mixed film and the normalized EL spectra of 26DCzPPy: B4PyPPM 26DCzPPy/B4PyPPM bilayer devices. (b) The transient PL decay of 26DCzPPy: B4PyPPM mixed film measured at 300 K. Figure S2. The current density-voltage-luminance characteristics of (a) blue and (b) orange co-host devices. The current efficiency-luminance-power efficiency characteristics of (c) blue and (d) orange co-host devices.
