# Supplementary material for: Management of Exciton Distribution for High-Performance Organic Light-Emitting Diodes Based on Interfacial Exciplex Architecture
Source: Molecules. 2023 Oct 11;28(20):7028. doi: 10.3390/molecules28207028 (PMC10609131; doi:10.3390/molecules28207028)
Supplement: Supplementary file 1 [file molecules-28-07028-s001.zip › molecules-2606202-supplementary.pdf]

Management of Exciton Distribution for High-Performance Organic Light-Emitting Diodes Based on Interfacial Exciplex Architecture

Ren Sheng [1,†], Cong Chen [1,†], Erdong Zhang [1], Wencheng Zhao [1], Jihua Tang [1], Duxu Yan [1], Zhengze Li [2] and Ping Chen [1,*]

1 Institute of Physics and Electronic Information, Yantai University, Yantai 264005, China; rensheng@ytu.edu.cn (R.S.); congchen@s.ytu.edu.cn (C.C.); erdongzhang@s.ytu.edu.cn (E.Z.); 18265892850@163.com (W.Z.); fanhuokua111@126.com (J.T.); yandx316@s.ytu.edu.com (D.Y.)

2 State Key Laboratory on Integrated Optoelectronics, College of Electronic Science & Engineering, Jilin University, Changchun 130012, China; 13091682837@163.com

* Correspondence: pingchen@jlu.edu.cn; Tel.: +86-1360-431-2567

† These authors contributed equally to this work.

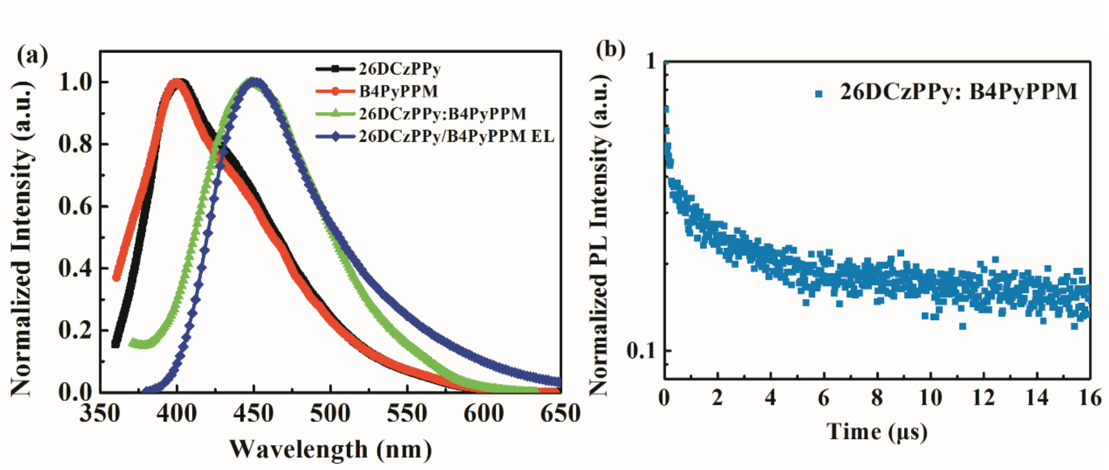

Figure S1. (a) The normalized PL spectra of 26DCzPPy, B4PyPPM and 26DCzPPy: B4PyPPM mixed film and the normalized EL spectra of 26DCzPPy: B4PyPPM 26DCzPPy/B4PyPPM bilayer devices. (b) The transient PL decay of 26DCzPPy: B4PyPPM mixed film measured at 300 K.

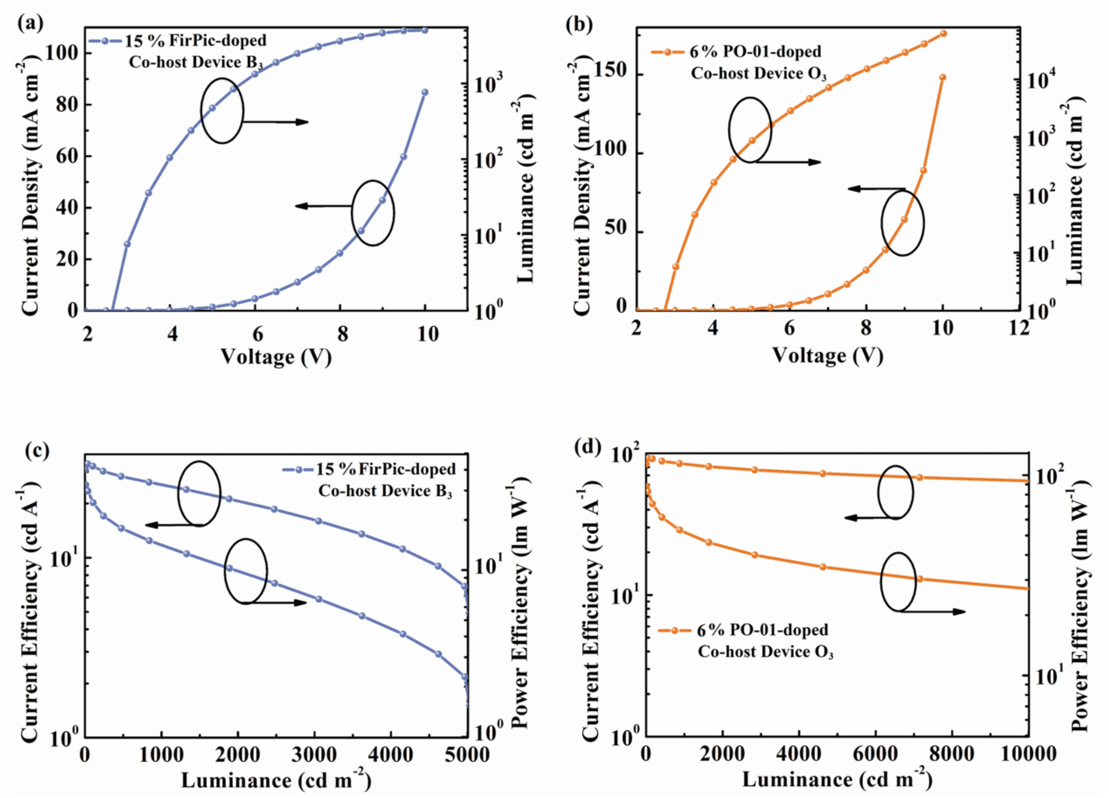

Figure S2. The current density-voltage-luminance characteristics of (a) blue and (b) orange co-host devices. The current efficiency-luminance-power efficiency characteristics of (c) blue and (d) orange co-host devices.
